# Spontaneous migration of a falling bullet in the cerebellum reveals the importance of intraoperative skull X-ray

**DOI:** 10.1093/jscr/rjab105

**Published:** 2021-04-19

**Authors:** Ali Hammed, Moufid Mahfoud, Alaa Sulaiman

**Affiliations:** Tishreen University Hospital, Department of Neurosurgery, Lattakia, Syria; Tishreen University Hospital, Department of Neurosurgery, Lattakia, Syria; Tishreen University Hospital, Department of Neurosurgery, Lattakia, Syria

**Keywords:** cranial gunshot wounds, falling bullet, migration, intraoperative X-ray

## Abstract

Cranial gunshot wounds (CGSWs) are the most lethal types of the cranial traumas and they are usually mortal. Falling bullets or gravitational bullets are the ones that move under the effect of the gravity force after the muzzle force diminished. CGSWs constitute a major clinical challenge for neurosurgeons dealing with trauma in both the military and civil experience. We report the case of a 21-year-old man with a falling bullet wound to the head. The decision of surgical treatment of a bullet injury is difficult if it is in close proximity to vital structures; removal of the bullet may cause significant neurological damage; however, migration can lead to a worsening of the neurological status of the patient. Before surgical removal of any intracranial bullet, as valuable information, it is recommended that a plain skull X-ray be obtained after final positioning of the head.

## INTRODUCTION

Cranial gunshot wounds (CGSWs) are the most lethal types of the cranial traumas and they are usually mortal. The mortality rate has been reported as ranging from 51 to 84% [1].

Falling bullets or gravitational bullets are the ones that move under the effect of the gravity force after the muzzle force diminished.

Low-velocity FB wounds have a vertical or high incident angle, even though the kinetic energy is very low compared with that of directly fired missiles.

These injuries are more serious because the possibility of fatal head wounds is very high [2].

Traumatic brain injury caused by a gunshot wound is a complex injury with a broad spectrum of symptoms and high rates of mortality and morbidity.

Low GCS scores, ventricular injuries and bihemispheric injuries are correlated with poor prognosis [3].

The frequency of gunshot injuries to the head is on the increase and it becomes an important public health problem. Migration of the retained bullet is rare and has been reported to occur in 0.06–4.2% of cases [4].

Migration of an intracranial bullet is a rare complication of gunshot injuries to the head. The time course for migration ranges from 2 days to 3 months [5].

Treatment of gunshot injuries is still debatable. The neurological status at the time of entry and location of the bullet often dictate the decision regarding surgical removal.

## CASE PRESENTATION

A 21-year-old male was admitted to the emergency unit after a falling bullet wound to the head.

In his physical examination, there was a single entry wound situated on the left side of the parietal bone, about 2 cm left of the midline.

Brain tissue was seen through the open wound. In the first neurological examination, the patient was confused and GCS at the time of admission was 14.

**Figure 1:**
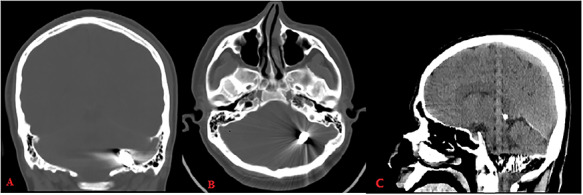
Computed tomography (bone window: **A**: coronal, **B**: axial) reveals metallic object located between left occipital bone and inferior side of left cerebellar hemisphere; CT (brain window **C**: sagittal) shows hemorrhage along its trajectory.

Pupils were equally reactive, and his vitals were stable. Cranial computerized tomography (CT) revealed a bone defect of 0.5 cm in the left parietal region and a metallic object located between left occipital bone and inferior side of left cerebellar hemisphere (Fig. 1).

The bullet had passed through the left parietal lobe. There was hemorrhage along its trajectory, but the metal artifacts obscured the damage. The entry wound was debrided and sutured.

Three days later, the patient showed new neurologic defects (Vomiting, headache and dizziness), so we decided to operate to remove the bullet.

**Figure 2:**
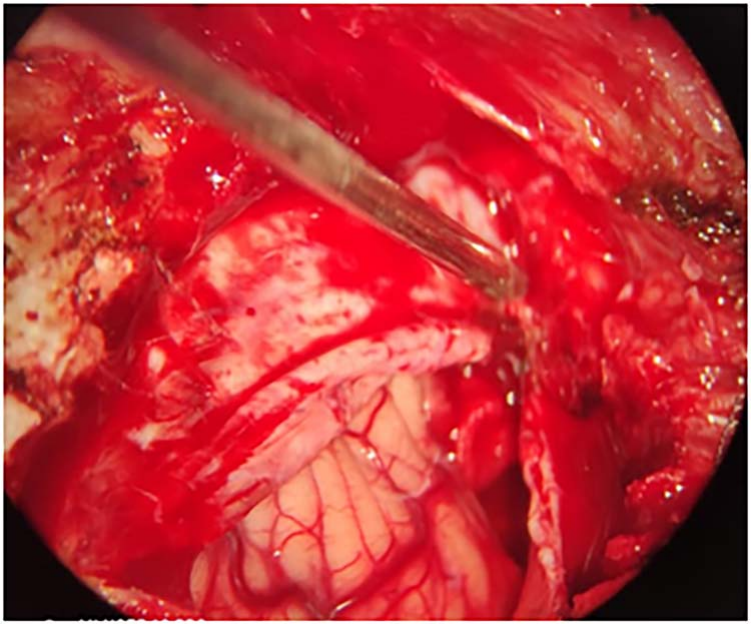
Intra-operation image shows the contused brain tissue which was caused by bullet in left cerebellar hemisphere.

The patient underwent a left unilateral retrosegmoid craniectomy in the park bench position. However, although the contused brain tissue was drained (Fig. 2), the projectile was not found in the suspected point.

**Figure 3:**
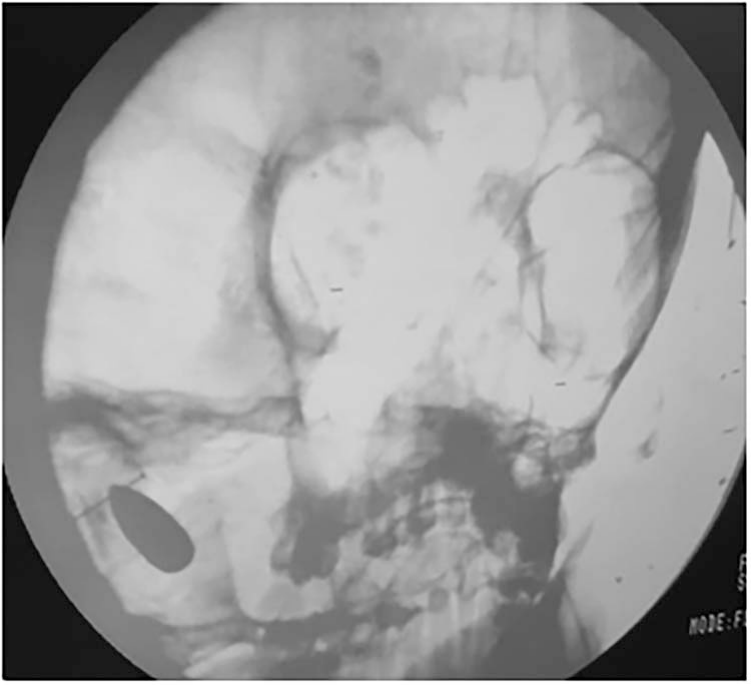
Intra-operation skull X-ray revealed that bullet had inversed and migrated upwards inside the left cerebellar hemisphere.

A plain skull X-ray was obtained and it revealed that the bullet had inversed and migrated upwards inside the left cerebellar hemisphere (Fig. 3).

Another maneuver was done and the bullet was removed.

The patient was discharged after 3 days without any neurologic deficit.

## DISCUSSION

CGSWs constitute a major clinical challenge for neurosurgeons dealing with trauma in both the military and civil experience.

Falling bullets or gravitational bullets are the ones that move under the effect of the gravity force after the muzzle force diminished.

The outcome of head injury from falling bullets depends on age, Glasgow coma score on admission, reactivity of the pupils, the missile course through the brain or base of the patency of basal cisterns skull as well as structures injured through its cerebral course.

There is no single common anatomical location of gunshot bullet in the brain. However, according to the literature, posterior fossa remains the most common location of migrated bullet [6].

Besides, the bullet fragment remaining in the skull causes many other secondary complications sooner. Common complications of retained intracranial foreign bodies consist of abscess formation, cerebrospinal fluid fistulas, post-traumatic epilepsy, hematomas and infection. Among them, the most important one is the irremovable bullet fragment or the increasing infection risk under the foreign bodies [7].

Intracranial infections are a feared complication of penetrating missile injuries to the brain. They occur in 3–17% of all cases, generally forming within 1–5 weeks after injury. The mortality rate of patients with these infections has been reported to exceed 50% [8].

Furthermore, another significant complication is the movement of the bullet in the head. This unforeseen complication was first introduced at 1916 by Vilvandré and Morgan [9].

Gravity, brain pulsation and tissue softening have been postulated as mechanisms for a migrating bullet fragment [7].

We believe that final operative positioning of the head according to patient position (park bench, prone or reverse trendelenburg) significantly influenced movement of the bullet.

Management of retained intracranial fragments remains controversial. The evidence supporting conservative brain debridement is Class III, as there are no controlled studies suggesting the efficacy of various degrees of debridement in preventing infection or the development of seizure disorders [10].

The decision of surgical treatment of a bullet injury is difficult if it is in close proximity to vital structures, removal of the bullet may cause significant neurological damage; however, migration can lead to a worsening of the neurological status of the patient.

## CONCLUSION

The spontaneous migration of bullets within the brain has been reported sporadically. Before surgical removal of any intracranial bullet, as valuable information, it is recommended that a plain skull X-ray be obtained after final positioning of the head.
